# The Digital Clock and Recall is More Equitable and Less Biased than the Mini‐Mental State Examination in Terms of Ethnicity

**DOI:** 10.1002/alz.089963

**Published:** 2025-01-03

**Authors:** Claudio Toro‐Serey, Ali Jannati, Russell Banks, John Showalter, David Bates, Sean Tobyne, Alvaro Pascual‐Leone

**Affiliations:** ^1^ Linus Health, Boston, MA USA; ^2^ Harvard Medical School, Boston, MA USA; ^3^ Michigan State University, East Landing, MI USA; ^4^ Hebrew SeniorLife, Boston, MA USA

## Abstract

**Background:**

Early detection of cognitive impairment is crucial for maximizing the benefits of disease‐modifying treatments for Alzheimer’s disease (AD). Brief, automatically‐scored digital cognitive assessments such as the Digital Clock and Recall (DCR) show promise in streamlining this early detection. However, wide adoption of such assessments in diverse populations requires evaluation of their demographic biases. Here, we compared the biases due to ethnicity, race, and education level between the DCR and the Mini‐Mental State Examination (MMSE).

**Method:**

We studied 706 primarily English‐speaking participants from Bio‐Hermes‐001 study (age mean±SD = 71.5±6.7; 58.9% female; years of education mean±SD = 15.4±2.7; 85.1% White; 9.3% Hispanic), classified a priori as cognitively unimpaired (CU; n = 360), mild cognitive impairment (MCI; n = 234), or probable Alzheimer’s dementia (pAD; n = 111) based on expert consensus and neuropsychological evaluation. We also studied 770 participants in a prescreening study for AD‐related clinical trials (age mean±SD = 68.8±13.2; 64% female; education = 15±2.6; 78% White; 20% Hispanic) including CU (n = 338), MCI (n = 178), and pAD (N = 254). For each dataset, bias was compared by bootstrapping two multiple linear regressions predicting either Z‐scaled DCR or MMSE scores using race, ethnicity, sex, education, and age as predictors. The bootstrapped demographic coefficients (i.e. mean differences) were compared between tests and significance was determined via 95% confidence intervals on these differences.

**Result:**

A larger ethnicity bias was observed for the MMSE than for the DCR, which was significant in the Bio‐Hermes (bias difference = 0.44 larger for MMSE, 95% CI = 0.12–0.75) but not the pre‐screener study (bias difference = 0.20 larger for MMSE, 95% CI = ‐0.32–0.74). We found significant differences between Hispanic and non‐Hispanic individuals only for MMSE in both datasets (Mann‐Whitney test, *p*<0.01). Bias for race and education was not significantly different between tests.

**Conclusion:**

Unlike the MMSE, the DCR is not influenced by ethnicity. Given the higher prevalence of cognitive impairment and greater risk of dementia in ethnic minorities (with Hispanics forming the largest minority group), the deployment of less biased assessments such as the DCR is important for more equitable cognitive screening.

